# Nurture-U student mental health longitudinal survey: a study protocol

**DOI:** 10.1136/bmjopen-2024-098413

**Published:** 2025-02-11

**Authors:** Ellen Marshall, Anne C Duffy, Samuel R Chamberlain, Jemima Dooley, Lucy Dorey, Liz Forty, Kevin A Matlock, Alexandra Newbold, Anthony Quinn, Sarah Rees, Lucy J Robinson, Kate E Saunders, Edward R Watkins

**Affiliations:** 1 Newcastle University, Newcastle upon Tyne, UK; 2 Psychiatry, Queens University, Kingston, Ontario, Canada; 3 Department of Psychiatry, University of Oxford, Oxford, UK; 4 Department of Psychiatry, University of Southampton, Southampton, UK; 5 Southern Health NHS Foundation Trust, Southampton, UK; 6 University of Exeter, Exeter, UK; 7 Cardiff University School of Medicine, Cardiff, UK; 8 Worcester College, University of Oxford, Oxford, UK; 9 Cardiff University, Cardiff, UK

**Keywords:** MENTAL HEALTH, Anxiety disorders, Depression & mood disorders

## Abstract

**Abstract:**

**Introduction:**

University life represents a critical period for young adults, providing opportunities for personal growth and development of coping skills but also posing significant mental health challenges. Recent trends indicate rising mental health concerns among university students, exacerbated by the COVID-19 pandemic and its aftermath. This study aims to address gaps in longitudinal data on student mental health in the UK and to identify risk and protective factors across diverse student populations.

**Methods and analysis:**

The current Nurture-U survey is developed from the U-Flourish biannual survey study piloted at Queen’s and Oxford universities in Canada and the UK, respectively. Nurture-U is a longitudinal survey study conducted at five UK universities, aiming to create a comprehensive data set from over 5000 students. The study will collect data at the start and completion of each academic year, using validated measures to assess well-being, mental health symptoms, lifestyle factors and access to support. Recruitment will target all students, with an emphasis on first-year students, to track their mental health trajectory from university entry through subsequent years.

**Ethics and dissemination:**

Ethical approval has been obtained from relevant committees at each participating university. Students will provide informed consent prior to participation, with risk messages and support information provided for those indicating self-harm or suicidal thoughts. Data will be de-identified and securely stored, with results disseminated through academic publications, social media and student engagement activities.

**STRENGTHS AND LIMITATIONS OF THIS STUDY**

- Large-scale, multisite study: The study’s extensive reach across five UK universities ensures a large-scale, diverse and representative sample of the student population.
- Robust data collection: Employing clinically validated instruments and covering a wide range of factors influencing mental health (including personal, family, environmental, psychological and lifestyle factors) enhances the reliability, validity and comprehensiveness of the data collected.
- Longitudinal design: Repeated measures allow for tracking mental health trajectories over time, providing insights into how student well-being evolves throughout university.
- Selection and engagement bias: There may be inherent biases in who chooses to participate which can vary by university, potentially affecting the representativeness and comparability of the findings.
- Self-reported data: Reliance on self-reported measures can introduce response biases and inaccuracies, particularly concerning sensitive topics like mental health.

## Introduction

 Entering university is an exciting time for students: establishing a lifestyle and asserting new-found independence present opportunities for personal growth, new experiences and developing vital coping skills. Nevertheless, the transition to university life often brings upheaval, requiring rapid adjustment and the expenditure of considerable personal resources.^1^ University can bring new opportunities and experiences and although these can be positive for many, they can place students at high risk of stress and mental health difficulties which can severely impact learning and engagement. This not only increases the likelihood of dropping out and underachievement but also contributes to differential attainment, where students with mental health conditions face significant barriers to academic progression and success. Previous research suggests that certain groups of students are disproportionately affected by poor mental health, exacerbating the attainment gap and leading to poorer career prospects and long-term disadvantage.^2 3^

Mental health difficulties are increasing among university students in the UK and across the Western world with anxiety, depression, self-harm and substance use problems being common.^4^,^8^ In the UK, between 2011 and 2021, there was a 450% increase in students declaring a mental health disability when applying for university.^9 10^ Additionally, the percentage of students saying they had experienced a mental health problem increased from 6% to 16% over a 6-year period from 2016/2017 to 2022/2023.^11^ The COVID-19 pandemic added to the student mental health burden, with further increases in anxiety, depression and sleep problems during peak lock-down periods^12 13^ due to learning remotely, worry for older family members, reduced socialising with peers and concern about the impact on academic study and overall grades.^14^ Unsurprisingly, university well-being services have also reported an increase in demand for services, with some reporting a doubling of the number of students accessing support.^10 15^ This trend has strained university resources and raised concerns about the scope of mental health support that universities should provide and how these connect with community-based and external services.^16^

Although the primary causes of the increasing incidence of mental health difficulties among university students are not fully understood, it is likely influenced by various factors. A systematic review identified that demographic characteristics, gender, family dynamics, childhood adversity, parental mental health, environmental conditions, lifestyle habits and psychological variables all play a role in shaping students’ mental health and well-being.^17^ More specifically, a large UK-based demographic study revealed that women, LGBTQ+individuals and students from lower socioeconomic backgrounds face heightened vulnerability to mental health challenges.^11^ An additional UK cohort study found that levels of psychological distress generally rise as young people transition from school and enter university.^18^ University students encounter various stressors spanning financial, academic and social domains that are specific to the university environment,^19^ with social relationships notably emerging as a primary stressor for students.^20^ Research has highlighted the significant impact of loneliness and social isolation on mental health, while a sense of belonging and robust support networks are linked to enhanced mental health and well-being.^21 22^ These findings align with broader meta-analyses exploring the relationship between social support and mental health in the general population.^23^

Student mental health has been recognised as a priority by a broad spectrum of stakeholders within the higher education sector, including researchers, universities and advocacy organisations such as Universities UK and Student Minds.^24 25^ In particular, the Student Mental Health Research Network have identified seven areas of need within student mental health research: epidemiology, causes and risk factors, academic factors and work-life balance, sense of belonging, intervention and services, mental health literacy and consequences. However, current research on student mental health in the UK, as elsewhere, is hindered by several key limitations.^26 27^ One major constraint is the lack of use of validated and clinically robust measures for assessing mental health difficulties among students—while there have been many surveys of students, few have used validated measures and most use bespoke internal measures (for exceptions see Auerbach *et al;*^4^ Auerbach *et al;*^5^ Duffy *et al*^16^). Another major limitation is the near-exclusive focus on cross-sectional surveys. As such, longitudinal studies, which would be essential for understanding the trajectory of mental health difficulties over time and identifying potential risk or protective factors, are scarce. Additionally, the absence of standardised measures across institutions and studies complicates comparisons and the generalisability of findings. Furthermore, while many studies have aimed to gain a snapshot of students’ levels of symptoms and well-being, few studies have examined in depth the association of symptoms with potential mechanisms of risk and resilience, including psychological and behavioural variables. Surveys of students have tended to be localised within a single institution or comprise very brief surveys offered across multiple institutions. Consequently, addressing these limitations by employing validated measures, conducting longitudinal studies, including measures of potential mechanisms and establishing standardised data collection methods across multiple and diverse institutions is crucial for advancing our understanding of student mental health and facilitating effective interventions and policies.

Given the increased reporting of mental health difficulties in students and the rise in demand for services, the need to address student mental health difficulties is both a moral imperative and a pragmatic necessity. This protocol outlines the methodology for a large-scale, multisite, longitudinal mental health survey study in students across five UK universities. This survey approach builds on the Canadian U-Flourish Student Well-Being and Academic Success Study survey piloted at the University of Oxford in 2019.^28^ The U-Flourish Study is a longitudinal survey that repeats twice in the academic year, which includes validated measures of symptoms, well-being, lifestyle, personality and cognitive factors, stigma and experience of university (see Goodday *et al*^28^ for full study protocol and King *et al*^8^ for baseline findings). The U-Flourish biannual survey has captured trends in two key areas: student mental health difficulties over the pandemic and trajectories of incoming undergraduate cohorts from entry to degree completion in Canadian universities (2018–2024) and at the University of Oxford (2018–2021). It has also examined associations between early and proximal psychological and social risk factors, student status and common mental health concerns^38 12^,^14 19 29^ as well as barriers and experience of help-seeking.^19 32^

### Aims

The present study, Nurture-U, aims to create a large, representative, longitudinal data set of over 5000 UK university students across five institutions (Exeter, Oxford, Newcastle, Southampton and Cardiff). This survey aims to gain a comprehensive understanding of students’ mental health burdens and associated factors as they transition to university and navigate university life. By assessing well-being, mental health, process mechanisms and access to support services biannually, the research focuses on how mental health needs and difficulties vary across diverse student groups (eg, gender, sexuality, socioeconomic background, ethnicity, prior mental health history) and over time. The study also seeks to identify barriers and facilitators to accessing support, with the ultimate goal of informing strategies to better support well-being throughout students’ university journeys.

## Methods and analysis

### Design

This is a prospective longitudinal survey occurring at two time points within each academic year, to allow for the investigation of factors that predict changes in mental health and well-being over the course of an academic year. The survey will be repeated across cohorts over three academic years, to allow for opportunities to increase sample size with new entrants, examine annual variations and track some students across multiple years of study.

Data collection for the baseline survey will take place at the start of the academic year for each university. This ranges from the third week in September (Exeter, Newcastle and Southampton) to the third week in October (Oxford). The follow-up survey will occur near the end of the term in spring (6 months later) and will be open for 3 weeks before university examinations.

#### Study setting and sites

The survey will take place at five UK universities: Cardiff University, University of Exeter, Newcastle University, University of Oxford and University of Southampton. These universities range across geography (Northeast to Southwest England, Wales) and in terms of being campus versus city-based. Currently, there are over 135 000 students (undergraduate and postgraduate) enrolled at these universities. The universities are ranked 1st–24th in the UK and 1st–177th in the world (Times Higher Education, 2023). All universities offer undergraduate and postgraduate courses and welcome both home and international students.

### Participants

Recruitment for the Nurture-U survey is open to all students (all years, undergraduate and postgraduate), with an emphasis on recruiting first-year students to enable monitoring of the student for the longest period throughout their university journey at four of the five universities (Cardiff, Exeter, Newcastle and Southampton). Consistent with the pre-existing U-Flourish study, Oxford is focusing solely on first-year undergraduate and graduate students. Only participants who completed the baseline (autumn) survey will be invited to take part in the follow-up (spring) survey. Recruitment is planned to last from Autumn (late September) 2022 through to Spring (April) 2025.

### Survey

#### Development of survey

The survey used in this study will be adapted from the U-Flourish survey, with careful consideration given to selecting measures that align with the specific aims of this research project and those that help to ensure data combining from both projects in the future. Supplementary measures were also integrated to address the primary goals of the Nurture-U project, informed by student feedback and preferences, with particular attention paid to pertinent topics such as the impact of COVID-19.

#### Survey content

The full core questionnaire and psychometric properties of each measure can be found in online supplemental material 1. The baseline questionnaire comprises validated measures over six sections (table 1): personal factors (eg, demographics), family factors (eg, parental mental health and early adversity), psychological and emotional health (eg, anxiety, depressive symptoms and suicide-related behaviour), lifestyle habits, mechanisms and behaviour (eg, frequency of alcohol, caffeine consumption, exercise and sleep) and supplementary questions (eg, pandemic anxiety) (see table 1 for measure names and time-points).

**Table 1:** Measures included in the Nurture-U survey

Construct	Measure (items)	Time-point
Section 1: personal factors		
Demographics	Student ID numberStudent email addressDisability*	Baseline and follow-up
Demographics	AgeGender identity^34^Sexual orientation^34^Ethnicity^34^Year of study and course length*Undergraduate or postgraduate*Faculty/division*Home/international student*Accommodation—on/off campus, private/university owned*	Baseline
Section 2: family factors		
Parental mental health	Family history of mental illness*	Baseline
Parental education	Parental highest level of education*	Baseline
Parental impact	Divorce^34^Death^34^Age of divorce and/or death of parent^34^†	Baseline
Adverse childhood experiences	Childhood Experience of Care and Abuse (CECA)^35^	Baseline
Section 3: psychological and emotional health		
Mental health history	Mental health or learning disability diagnosis*Age of diagnosis*Previous mental health treatment*	Baseline
Psychosis	Youth Psychosis At Risk Questionnaire 2-item (YPARQ-2)^36^	Baseline and follow-up
Lifetime suicidality	Columbia Suicide Severity Rating Scale (C-SSRS)^37^	Baseline and follow-up
Current mental health	Mental health rating and functioning (day-to-day and school)Current mental health treatment	Baseline and follow-up
Well-being	Warwick Edinburgh Mental Well-being Scale 7-item (WEMWBS-7)^38^	Baseline and follow-up
Anxiety	Generalised Anxiety Disorder Scale 7-item (GAD-7)^39^	Baseline and follow-up
Depression	Patient Health Questionnaire 9-item (PHQ-9)^40^	Baseline and follow-up
Eating behaviour	Structured Clinical Interview for DSM-5 (SCID-5)—Screening questions for eating disorders^41^Sick, Control, One, Fat, Food (SCOFF) questionnaire^42^	Baseline and follow-up
Emotional clarity	Emotional Clarity—Difficulties in Emotion Regulation Scale 18-item (DERS-18)—Emotional Clarity Subscale^43^	Baseline and follow-up
Section 4: lifestyle, habits, mechanisms and behaviour		
Loneliness	UCLA Loneliness Scale 8-item (UCLALS-8)^44^	Baseline and follow-up
Self-compassion/self-care	Self-Compassion Scale Short Form (SCS-SF)—Self-Care Subscale^45^	Baseline and follow-up
Rumination/brooding	Ruminative Responses Scale 5-item (RRS-22)—Brooding Subscale^46^	Baseline and follow-up
Resilience	Resilience scale for Adolescents (READ)—Social Resources Subscale^47^Brief Resilience Scale (BRS)^48^	Baseline and follow-up
Response to stress	Cognitive and Behavioural Response to Stress Scale (CB-RSS)—2-item: Cognitive Frequency and Helpfulness and Behavioural Frequency and Helpfulness^49^	Baseline and follow-up
Alcohol use	Alcohol Use Disorders Identification Test for Consumption 3-item (AUDIT-C)^50^	Baseline and follow-up
Substance use	Frequency of substance use^34^	Baseline and follow-up
Substance use concern	Concern from others on drug/alcohol use*	Baseline and follow-up
Lifestyle factors	Exercise, smoking, caffeine, self-care frequency*	Baseline and follow-up
Stress	Perceived Stress Scale 4-item (PSS-4)^51^	Baseline and follow-up
Sleep behaviour	Sleep Condition Indicator 8-item (SCI)^52^	Baseline and follow-up
Physical health	Physical health conditions*	Baseline and follow-up
BMI	Height and weight	Baseline
Section 5: supplementary		
COVID questions	Pandemic Anxiety Scale (PAS)—Consequence and disease anxiety subscales^53^	Baseline
Section 6: academic connectedness and seeking support		
University connectedness	College Student Subjective Well-being Questionnaire (CSSWQ)—School Connectedness Subscale (with researcher questions added)^54^	Follow-up
Barriers to accessing care	Barriers to care checklist (BCC)^55^Barriers to Access to Care Evaluation scale (BACE)^56^	Follow-up
Student stress	Post-Secondary Student Stressors Index (PSSI)—Modified^57^	Follow-up
Access to support	Accessing mental health support in academic year*Experience of accessing support‡	Follow-up

* Single-item questions created by the research team.

† The items from the WHMO-ICCS web questionnaire^34^ initially adapted for Canada, were further adapted for cultural appropriateness in the UK and to align with UK census categories.

‡ Multi-item questions created by the research team.

BMIbody mass indexDSM-5Diagnostic and Statistical Manual of Mental Disorders 5UCLAUniversity of California Los AngelesWHM-ICSWorld Mental Health - International College Student

The follow-up questionnaire will include the same measures as the baseline survey, with the addition of measures of support-seeking and student life satisfaction (section six). These measures only make sense to be included after students have been present at university for a significant amount of time and have the time to experience university life and the support available to them. Only demographics that could change through the academic year will be collected at follow-up (eg, disability, mental health diagnosis), with background and personal history demographics collected at the winter survey not repeated.

### Recruitment

The recruitment strategy for the baseline survey will focus on four main areas: mass communication, on-campus promotion, lecture shout-outs and social media. Each university will adopt tailored strategies. See table 2 for details.

**Table 2:** Planned recruitment for baseline survey at each university site

	Exeter	Newcastle	Southampton	Cardiff	Oxford
Mass communication	✓	✓			✓
Social media	✓	✓	✓	✓	✓
On campus (stalls and posters)	✓	✓	✓	✓	
Lecture shout outs	✓	✓	✓	✓

1. Mass (university) communication to students:

At Exeter, Newcastle and Oxford emails containing study information and survey links will be sent from high authority figures or university official accounts. Additionally, survey links will be posted on virtual student noticeboards, learning system noticeboards and university newsletters at Newcastle and Exeter.

1. On-campus promotion

On-campus recruitment techniques include stalls with refreshments/promotional merchandise, posters, leaflets and digital screen adverts around campus.

1. Lecture shout-outs

Four universities will conduct lecture shout-outs in first-year lectures, varying by format and size. A QR code linking to the survey will be provided during breaks or as part of a talk by a member of the research team. Follow-up emails will be sent to lecturers to distribute the survey link.

1. Social media

The survey will be promoted via Nurture-U’s Instagram and Facebook pages through paid adverts targeted to students (ages 17–26) within a 2 km of each campus. Adverts will be individualised for Exeter, Newcastle and Southampton. At Oxford, adverts will be promoted through college X (formerly Twitter) accounts only due to its collegiate setup.

For the follow-up survey, all sites will email the survey link to the students who had completed the baseline, along with reminder emails during the period that the survey is open.

### Incentives

Incentives for survey completion will differ across sites in accordance with local policies and ethical approval. Participants will be entered into raffles for various prizes, including cash rewards, shopping vouchers, iPads and university-branded clothing.

### Procedure

Students will complete the survey online using preprogrammed survey software (eg, Qualtrics, RedCap), with identical questions set and administered across each site, with a common data dictionary and metadata. On accessing the survey through one of the various recruitment channels, participants will be presented with an information sheet outlining key details of the study including: the name of the study, study funder, investigators and the aims of the research. Students must provide informed consent online to participate in the study. They cannot proceed with the survey unless they provide informed consent and their student email address. For participants indicating current thoughts of suicide or self-harm within their answers, risk messages and support information are provided on screen. The baseline survey takes approximately 20 min to complete, while the follow-up survey takes about 15 min.

### Public and patient involvement

The full outline of public and patient involvement is published elsewhere.^33^ Briefly, the project established the Nurture-U Student Advisory Group (SAG) which includes over 100 student members from all participating universities. SAG members have actively contributed to all stages of the Nurture-U project and will continue to provide guidance throughout the survey study. Although the Nurture-U survey was initially developed by the research team based on the U-Flourish survey, SAG members will have the opportunity to propose additions or modifications to the survey measures.

### Data linkage

A data-sharing agreement has been signed by all universities within the consortium. Data will be managed and processed at each university and backed up locally on secure servers, accessible only by team members and managed in line with all current data protection regulations. To link the data from all sites, de-identified data from each site will be sent to the University of Oxford for analysis where all files will be stored using a secure service and will only be accessible to the research team. Research data will be kept with the strictest confidence in line with the EU General Data Protection Regulation (2018). Anonymised and de-identified data will be available via a repository on completion of data collection and the primary study analyses.

### Planned analysis

One of the aims of the project is to build a large data set that could potentially be used to answer a range of different questions. In total, the study aims to recruit a sample of 5000 students across five UK universities. A large sample size allows for robust subgroup analyses, which are crucial for understanding the unique experiences and needs of different student populations, including international students, LGBTQ+individuals and those from lower socioeconomic backgrounds.

No data analysis has begun. The first analysis will characterise student mental health on entry to university and track how it changes over the academic year and through the university journey (entry to graduation). A combination of descriptive statistics, inferential statistics and advanced multivariate techniques will be used. Descriptive statistics will provide an overview of the sample characteristics and prevalence rates of mental health issues. Inferential statistics, including t-tests and χ² tests, will be used to compare mental health outcomes across different demographic groups. Advanced multivariate techniques, such as linear and logistic regression analyses, will identify predictors of mental health outcomes and evaluate the impact of various factors on student well-being. Finally, longitudinal data analysis methods will be used to examine changes in mental health over time and the factors influencing these trajectories.

### Ethics and dissemination

Ethics was obtained at each university site: Exeter (FHLS Psychology Research Ethics Committee, 519175), Cardiff (Wales Research Ethics Committee, IRAS reference: 155838, REC reference: 16/WA/0323), Newcastle (Faculty of Medical Sciences Research Ethics Committee, 24735/2022), Oxford (Medical Sciences Interdivisional Research Ethics Committee, Ref. R60998), Southampton (Faculty of Medicine Ethics Board, ERGO 73178). At all sites, participants will receive an information sheet and are required to provide informed consent before completing the survey. All participants will have to consent to be contacted for follow-up surveys.

The findings of the survey will be communicated using a comprehensive dissemination strategy. The strategy will use various forms of media to reach a diverse range of stakeholder groups and individuals, at the local, national and international levels. This will include the use of academic media (ie, peer-reviewed journal articles, national and international conference presentations), social media (ie, Instagram, X), print media (ie, British Psychological Society blog), funding meetings and student engagement activities (ie, forums at each university site, student conferences).

## Discussion

The Nurture-U student mental health survey addresses the critical gap in understanding how student well-being and mental health needs vary across diverse groups (eg, gender, sexuality, socioeconomic background, ethnicity, prior mental health history) and over time. This large-scale, multisite survey study will provide comprehensive insights into the mental health and well-being of UK students and identify barriers and facilitators to accessing support for diverse student groups. By using validated measures and conducting longitudinal assessments, the study aims to identify key risk and protective factors and inform interventions and policies to enhance student mental health outcomes. The survey findings are poised to significantly advance the understanding of student mental health and well-being. By identifying specific stressors and protective factors relevant to diverse student populations, universities will be able to use this information to tailor a wide range of support services, including mental health, academic, disability, residential and personal tutoring services, to better meet the needs of their students. The longitudinal nature of the study will provide crucial insights into how mental health and well-being evolve over the course of university life which is essential for developing inclusive preventative strategies and early interventions aimed at enabling all students to thrive.

## supplementary material

10.1136/bmjopen-2024-098413 online supplemental material 1
