# Supplementary material for: Nurture-U student mental health longitudinal survey: a study protocol
Source: BMJ Open. 2025 Feb 11;15(2):e098413. doi: 10.1136/bmjopen-2024-098413 (PMC11815430; doi:10.1136/bmjopen-2024-098413)
Supplement: online supplemental material 1 [file bmjopen-15-2-s001.docx]

**Supplementary Material 1**

| **Childhood Experience of Care and Abuse (CECA)**  The Childhood Experience of Care and Abuse (CECA; Bilfucio et al., 1994) assesses experiences of physical and sexual abuse during childhood. In the Nurture-U study, four items were used to assess these experiences: 1) physical abuse, 2) emotional abuse, 3) sexual abuse, which were adapted from the original measure, and 4) bullying, with an additional question devised by the research team ("When you were a child or a teenager, were you physically or verbally bullied or teased very badly by peers?"). The full CECA has demonstrated satisfactory reliability and validity as a self-report tool for assessing adverse childhood experiences (Bilfucio et al., 2005). However, specific psychometric details for the individual items used in the Nurture-U study have not been reported.  Variable type: Binary (presence/absence of abuse) |
| --- |
| **Youth Psychosis At Risk Questionnaire 2-item (YPARQ-2)**  The YPARQ-2 is a brief, two-item screening tool designed to detect early indicators of psychosis in adolescents. The full measure, including a longer version, has shown excellent internal consistency (α = 0.94) in adolescent non-clinical samples (Fonseca-Pedrero et al., 2016). The YPARQ-2 specifically has demonstrated strong psychometric properties, achieving a specificity of 98% and sensitivity of 53% in identifying clinically significant symptoms in adolescent and student populations (Phalen et al., 2019).  A clinical cut-off score of 2 (both questions answered "yes" regarding symptoms such as hallucinations and hearing voices) further enhances specificity, supporting its use as a targeted tool for identifying youth potentially at risk for psychosis.  Variable type: Binary using clinical cut off |
| **Columbia Suicide Severity Rating Scale (C-SSRS)**  The Columbia Suicide Severity Rating Scale (C-SSRS; Posner et al., 2011) measures the severity of suicide-related thoughts and behaviours, including passive and active thoughts, suicide attempts, and non-suicidal self-harm. For the Nurture-U survey, four items from the C-SSRS were included: 1) wishes of death or not waking up, 2) thoughts about ending one’s life, 3) lifetime suicide attempts, and 4) self-harm without suicidal intent. Participants responded with yes or no to each item. The C-SSRS has shown strong convergent and divergent validity in adolescent clinical and non-clinical samples, demonstrating its ability to effectively assess suicide risk and related behaviours (Posner et al., 2011; Azcurra, 2017).  Variable type: Binary (presence/absence of suicidal thoughts/behaviours) |
| **Warwick Edinburgh Mental Wellbeing Scale 7-item (WEMWBS-7)**  The Warwick Edinburgh Mental Wellbeing Scale - Short Version (WEMWBS-7; Clarke et al., 2007) is a 7-item tool designed to measure subjective wellbeing. Extensive research has validated its psychometric properties, reporting Cronbach's alpha score of 0.89 (student sample) and 0.91 (population sample) (Tennant et al., 2007). In the short version, total scores must be transformed for comparison with the longer versions of the scale. According to the original authors, a score of 19.5 or lower indicates 'low wellbeing', while a score of 27.5 or higher suggests 'high wellbeing'.  Variable type: Continuous total score and binary using clinical cut off |
| **Generalised Anxiety Disorder Scale 7-item (GAD-7)**  The Generalised Anxiety Disorder Scale 7-item (GAD-7) is a 7-item screening tool used to assess an individual’s severity of anxiety symptoms over the past two weeks. Respondents rate their experiences on a 4-point Likert scale ranging from 0 (not at all) to 3 (nearly every day). The GAD-7 has demonstrated excellent internal consistency (α = 0.89) and strong test-retest reliability in adult samples (Spitzer et al., 2006; Löwe et al., 2008). It also exhibits good convergent and divergent validity, correlating well with other measures of anxiety and depression.  The GAD-7 can be used both as a continuous measure (total score) and as a binary measure using clinical cut-offs, where a score of 10 or higher is typically used to identify clinically significant anxiety symptoms.  Variable type: Continuous (total score) and binary (clinical cut-offs) |
| **Patient Health Questionnaire 9-item (PHQ-9)**  The Patient Health Questionnaire 9-item (PHQ-9; Kroenke et al., 2001) is a 9-item tool used to assess depressive symptoms over the past two weeks. Respondents rate each item on a 4-point Likert scale ranging from 0 (not at all) to 3 (nearly every day). The PHQ-9 demonstrates excellent internal consistency (α > 0.85) and strong test-retest reliability (r = 0.87) in university student and primary care patient populations (Kroenke et al., 2001; Richardson et al., 2010; Zhang et al., 2013). It also shows good criterion and construct validity in both student and adult primary care patients (Levis et al., 2019; Kroenke et al., 2001).  The PHQ-9 can be used as a continuous measure (total score) and as a binary measure using clinical cut-offs, with a score of 10 or higher indicating clinically significant depressive symptoms (Levis et al., 2019).  Variable type: Continuous (total score) and binary (clinical cut-off) |
| **Structured Clinical Interview for DSM-5 (SCID-5)**  Two questions from the Structured Clinical Interview for DSM-5 (SCID-5) were used to screen for eating disorders:   - SCID-5 screening item for Anorexia Nervosa - *Recently, have you had a time when you weighed much less than other people thought you ought to weigh?* - SCID-5 screening item for Bulimia Nervosa and Binge Eating Disorder - *Recently, have you had eating binges, that is, times when you couldn’t resist eating a lot of food or stop eating once you started?*   Participants respond to these items with yes or no.  Variable type: Binary |
| **Sick, Control, One, Fat, Food (SCOFF) questionnaire**  Participants who indicated disordered eating by answering ‘yes’ to either of the eating disorder screening items (above) were then asked to complete the Sick, Control, One, Fat, Food (SCOFF) questionnaire.  The SCOFF is a brief, five-item questionnaire developed to screen for potential eating disorders, including anorexia nervosa and bulimia nervosa (Morgan et al., 2000). A threshold score of two or more positive (yes) responses is recommended by the original authors as indicative of a likely case. Research within university populations (mixed male and female samples) has demonstrated that this cut-off achieves a sensitivity of 53% and a specificity of 93%, with positive and negative predictive values of 67% and 89%, respectively (Parker et al., 2005). Additional findings suggest that raising the threshold to three positive responses results in a higher positive likelihood ratio of 11, compared to 6 when using the two-response cut-off (Cotton et al., 2003). For the Nurture-U study, the recommended threshold of two or more positive responses was utilised to identify individuals at risk for eating disorders.  Variable type: Continuous total score and binary cut off (2 or more = likely case of anorexia nervosa or bulimia) |
| **Difficulties in Emotion Regulation Scale 18-item (DERS-18) - Emotional Clarity Subscale**  The DERS-18 (Difficulties in Emotion Regulation Scale – 18; Victor & Klonsky, 2016) is an 18-item questionnaire adapted from the original 36-item version (Gratz & Roemer, 2004) to assess respondents' ability to regulate their emotions. For the Nurture-U survey, only the emotional clarity subscale was included, with two reverse-coded items removed for simplicity. Participants rate how often each statement applies to them on a five-point Likert scale, ranging from 1 (almost never) to 5 (almost always). The statements are: “I have no idea how I am feeling,” “I have difficulty making sense out of my feelings,” and “I am confused about how I feel.” The total score for the subscale is calculated by averaging the scores of the items, with higher scores indicating greater emotional dysregulation.  The DERS-18 has demonstrated strong psychometric properties across diverse samples, including high school students, university students, and adolescents in psychiatric settings. Internal consistency for the overall scale is excellent, with a Cronbach’s alpha of .91 in a combined sample (Victor & Klonsky, 2016).  Variable type: Continuous subscale score |
| **UCLA Loneliness Scale 8-item (UCLALS-8)**  A modified 4-item version of the original 8-item UCLA Loneliness Scale (Roberts et al., 1993) was used in the Nurture-U survey. The original authors noted that, although the 8-item scale appears to have a two-dimensional structure, this is largely due to the use of both negatively and positively worded items to counter response bias. They argue that the strong correlation between the two factors and the total scale, along with the consistent patterns of association with external variables, suggest that positive and negative items reflect the same underlying factor. Therefore, four reverse-coded items for brevity were excluded for the Nurture-U survey. The total score is calculated by summing all items, with higher scores indicating greater loneliness. The psychometric properties of the original 8-item measure have been extensively studied, demonstrating acceptable validity and reliability, with high internal consistency (alpha>0.80) reported in studies involving university student populations (Dogan et al., 2011; Hughes et al., 2004; Lasgaard, 2007).  Variable type: Continuous total score |
| **Self-Compassion Scale Short Form (SCS-SF) - Self-Care Subscale**  A six-item measure was used to assess the degree of self-compassion exhibited by participants. Respondents rated how often they engaged in specific behaviours (e.g., “I try to see my failings as part of the human condition”) on a five-point scale ranging from 1 (almost never) to 5 (almost always). The full Self-Compassion Scale Short Form Scale (SCS-SF; Raes et al., 2010) consists of twelve items divided into two subscales (six items each). For the Nurture-U survey, only the self-care subscale was used, which assesses participants’ levels of self-compassion, particularly in terms of tenderness, patience, and empathy towards themselves.  The total score for the self-care subscale is calculated by averaging the six items, with higher scores reflecting greater levels of self-care and self-compassion. In the original validation study of the short-form scale, the mean score for the self-care subscale was 3.11 (SD = 0.76; Raes et al., 2010). The full scale, encompassing both self-care and self-compassion subscales, has shown strong internal consistency in UK samples, with Cronbach’s alphas ranging from 0.84 to 0.92 (Ivtzan et al., 2018; Kotera et al., 2019). For the self-care subscale specifically, a study involving university students reported an internal consistency estimate of 0.79 (Hayes et al., 2016).  Variable type: Continuous total score |
| **Ruminative Responses Scale (RRS-10) - Brooding Subscale**  The Ruminative Responses Scale (RRS; Treynor et al., 2003) is a self-report tool designed to assess responses to depressive mood. The original scale contains 22 items across three factors: Depression, Brooding, and Reflection. The RRS-10, a shortened 10-item version of the RRS, excludes the Depression subscale while retaining the Brooding and Reflection subscales. For the Nurture-U study, the Brooding subscale was used, which included five items from the original RRS-10. Each item is rated on a 4-point Likert scale from 1 (never) to 4 (always). Total scores on the Brooding subscale range from 5 to 20, with higher scores indicating a greater tendency toward ruminative brooding. The RRS-10 has demonstrated strong psychometric properties, including high internal consistency and test-retest reliability in general populations (Extremera & Fernández-Berrocal, 2006; Lee & Kim, 2014; Schoofs et al., 2010; Treynor et al., 2003) and student samples (e.g., α = 0.82; He et al., 2021). Specific to the Brooding subscale, He et al. (2021) reported good internal consistency within a student population.  Variable type: Continuous total score |
| **Resilience scale for Adolescents (READ) - Social Resources Subscale**  The Social Resources subscale of the Resilience Scale for Adolescents (READ) assesses adolescents’ perceived social support from family and friends. This subscale includes items that ask respondents to rate the frequency of their social support experiences (e.g., “I have someone who will listen to me”) on a five-point scale ranging from 1 (strongly disagree) to 5 (strongly agree). Higher scores indicate greater perceived social resources and support. Scores are calculated by averaging responses across the items. In validation studies, the Social Resources subscale has demonstrated strong internal consistency, with Cronbach’s alpha values typically above 0.70 (Hjemdal et al., 2006; Von Soest et al., 2010). The READ has been widely used in adolescent populations across different cultural contexts, with the Social Resources subscale reliably measuring social resources as a resilience factor (Anyan et al., 2021; Hjemdal et al., 2006; Janousch et al., 2020).  Variable type: Continuous total score |
| **Brief Resilience Scale (BRS)**  The Brief Resilience Scale (BRS; Smith et al., 2008) is a six-item measure that assesses an individual’s ability to recover from stress. Respondents rate statements like “I tend to bounce back quickly after hard times” on a five-point scale, ranging from 1 (strongly disagree) to 5 (strongly agree). The BRS is designed to measure resilience as a process of recovery, focusing on an individual's capacity to "bounce back" rather than enduring adversity without change (Smith et al., 2008).  Total scores are calculated by averaging responses, with higher scores indicating greater resilience. The BRS has shown strong internal consistency across various populations, with Cronbach’s alpha values typically ranging from 0.80 to 0.91 (Smith et al., 2013; Rodríguez-Rey et al., 2016). It has also demonstrated good convergent and discriminant validity, showing positive correlations with measures of optimism and well-being, and negative correlations with depression and anxiety scales (Smith et al., 2013; Rodríguez-Rey et al., 2016). The BRS has been widely used in both clinical and general populations, providing a reliable and efficient measure of resilience across cultures.  Variable type: Continuous |
| **Cognitive and Behavioural Response to Stress Scale (CB-RSS) - 2-item: Cognitive Frequency and Helpfulness and Behavioural Frequency and Helpfulness**  The Cognitive and Behavioural Response to Stress Scale (CB-RSS; Miner et al., 2015) includes two 2-item subscales that assess cognitive and behavioural responses to stress: Cognitive Frequency and Helpfulness and Behavioural Frequency and Helpfulness. Each subscale evaluates how frequently adolescents use cognitive and behavioural strategies for dealing with stress and how helpful they perceive these to be. The Cognitive Frequency and Helpfulness subscale asks respondents to rate the frequency of thoughts they have in response to stress on a Likert scale (e.g., “I try to think positively about the stressful situation”) and their perceived helpfulness. The Behavioural Frequency and Helpfulness subscale similarly assesses how often they engage in coping behaviours (e.g., “I engage in activities to take my mind off the stress”) and the helpfulness of those behaviours. Scores are calculated by averaging the responses to the items within each subscale. Studies have found the CB-RSS to demonstrate strong internal consistency, with Cronbach’s alpha coefficients typically ranging from 0.70 to 0.90 for both subscales (Miner et al., 2015; Meulenbeek et al., 2017; Keane et al., 2020)  Variable type: Continuous |
| **Alcohol Use Disorders Identification Test for Consumption 3-item (AUDIT-C)**  The Alcohol Use Disorders Identification Test for Consumption (AUDIT-C; Bush et al., 1998) is a brief 3-item screening tool designed to assess the quantity and frequency of alcohol consumption as well as the presence of potential alcohol-related problems. The three items ask respondents to report how often they drink, the amount consumed on a typical occasion, and how often they have had six or more drinks on one occasion. Responses are rated on a scale ranging from 0 (never) to 4 (daily or almost daily), with higher scores suggesting higher levels of alcohol consumption and greater risk for alcohol use disorders. The AUDIT-C has shown strong psychometric properties, with good internal consistency (Cronbach's alpha values typically ranging from 0.70 to 0.90) across diverse populations, including both general populations and specific groups, such as college students and primary care settings (Bush et al., 1998; Reinert & Allen, 2007). It has demonstrated validity in identifying individuals at risk for alcohol misuse and alcohol use disorders.  Variable type: Continuous total score |
| **Perceived Stress Scale 4-item (PSS-4)**  The Perceived Stress Scale 4-item (PSS-4; Cohen et al., 1983) is a brief screening tool designed to assess perceived stress by asking individuals how frequently they encounter stress-related feelings over the past month. The four items ask participants to rate how often they felt overwhelmed by difficulties, how often they felt they could not control important things in their life, how often they felt nervous and stressed, and how often they felt confident about their ability to handle personal problems. Responses are scored on a 5-point Likert scale from 0 (never) to 4 (very often), with higher scores indicating greater perceived stress. The PSS-4 has shown strong psychometric properties, with acceptable internal consistency (Cronbach's alpha values typically around 0.60 to 0.82) across diverse populations, including both general populations and specific groups, such as university students (Lee, 2012).  Variable type: Continuous total score |
| **Sleep Condition Indicator 8-item (SCI)**  The Sleep Condition Indicator (SCI; Espie et al., 2014) is an 8-item measure designed to screen for insomnia symptoms based on DSM-5 criteria. It evaluates sleep quality, continuity, and perceived impact of sleep issues on daily life, asking respondents to reflect on their sleep over the past month. Each item is scored on a 5-point Likert scale, with scores ranging from 0 to 32, where higher scores indicate better sleep quality and lower risk of clinical insomnia. The SCI has been shown to have good internal consistency, with Cronbach's alpha values reported between 0.86 and 0.90 across various populations, including adults and adolescents (Espie et al., 2014; Cappuccio et al., 2018). Test-retest reliability is also strong, indicating stability over time. The SCI’s construct validity is well-supported, with significant correlations found between SCI scores and related measures of sleep quality and mental health, such as the Insomnia Severity Index (ISI) and Pittsburgh Sleep Quality Index (PSQI) (Espie et al., 2014).  **Variable type**: Continuous |
| **Pandemic Anxiety Scale (PAS) – Consequence and disease anxiety subscales**  The Pandemic Anxiety Scale (PAS) is a measure created to assess anxiety specifically related to a pandemic, with a focus on two subscales: Consequence Anxiety and Disease Anxiety. The Consequence Anxiety subscale evaluates anxiety surrounding the impact of a pandemic on one’s life, while the Disease Anxiety subscale measures anxiety regarding infection and illness. Respondents rate each item on a 5-point Likert scale from 0 (never) to 4 (always), with higher scores indicating higher levels of pandemic-related anxiety. The PAS total score is continuous, with distinct scores for each subscale. Psychometric studies of the PAS have shown strong internal consistency, with Cronbach’s alpha values typically reported between 0.80 and 0.90 for both subscales across various populations (Taylor et al., 2020; Wheaton et al., 2021).  Variable type: Continuous |
| **College Student Subjective Wellbeing Questionnaire (CSSWQ) - School Connectedness Subscale *(with researcher questions added)***  The College Student Subjective Wellbeing Questionnaire (CSSWQ; Renshaw, 2022) is a 16-item tool designed to assess students' satisfaction with their university experience, with the School Connectedness subscale focusing on students’ sense of belonging and engagement within the school environment. Responses are measured on a 7-point Likert scale, from 0 (strongly disagree) to 6 (strongly agree), with higher scores indicating greater subjective wellbeing related to school connectedness. Three additional items were added to capture experiences related to lived experience of diversity (“I have felt insulted or threatened based on my cultural/ethnic background at this university”, “I feel that this university honours diversity and internationalism” and “I have felt insulted or threatened based on my sexuality/gender identity”). The first and last of these items are reverse-scored.  Psychometric evaluation of the CSSWQ (without the additional 3 items) has demonstrated strong reliability and validity. For internal consistency, studies report Cronbach alphas ranging between 0.75 and 0.92 in adolescent and college student populations (Renshaw & Bolognino, 2016; Dixon et al., 2020).  Variable type: continuous total score |
| **Barriers to care checklist (BCC)**  12-item attitudinal and 6-item practical subscales from the Barriers to Care Checklist (Vanheusden et al., 2020) were used in the Nurture-U survey. These subscales assess attitudes and practical barriers towards accessing and receiving mental health care. Psychometrics of this checklist have not been formally evaluated.  Variable type: Checklist, no scoring |
| **Barriers to Access to Care Evaluation scale (BACE)**  The Barriers to Access to Care Evaluation (BACE; Clement et al., 2012) scale is a tool designed to assess perceived barriers to accessing mental health services, with a particular focus on stigma-related obstacles. In the Nurture-U survey, a 9-item stigma subscale from the BACE was used to measure the impact of stigma on individuals’ likelihood of seeking mental health support. Participants rate each item on a 4-point scale ranging from 0 (not at all) to 3 (always or almost always), and items are averaged for the overall score. Higher scores indicate stronger stigma-related barriers. The BACE has demonstrated robust psychometric properties. The stigma subscale shows strong internal consistency, with Cronbach’s alpha reported at 0.89 (Clement et al., 2012). Additionally, it exhibits satisfactory test-retest reliability over a two-week period, with weighted kappa values between 0.61 and 0.81 (Clement et al., 2012; Andrade et al., 2014).  Variable type: Continuous |
| **Post-Secondary Student Stressors Index (PSSI) – Modified**  The Post-Secondary Student Stressors Index (PSSI; Linden & Stuart, 2019) is a 46-item measure designed to assess stressors in post-secondary students across five domains: academics, learning environment, campus culture, interpersonal relationships, and personal challenges. Items are rated on their severity and frequency. In the Nurture-U study, the scale was condensed to 18 items, with some rewording, reflecting the original authors’ recommendation to treat each item as an individual causal indicator, as subscales were not viable. Psychometric testing shows strong test-retest reliability (rs = 0.78) and good construct validity, with moderate correlations to the PSS-10 and K10. Content validity was supported by extensive student input. Total scores are calculated by summing the number of items with a score of one or above, indicating some level of stress.  Variable type: Continuous. |

**References**

Andrade LH, Alonso J, Mneimneh Z, Wells JE, Al-Hamzawi A, Borges G, et al. Barriers to mental health treatment: Results from the WHO World Mental Health surveys. Psychol Med. 2014;44(6):1303-17. https://doi.org/10.1017/S0033291713001943

Anyan F, Morote R, Las Hayas C, Gabrielli S, Hjemdal O. Measuring resilience across participating regions in the UPRIGHT EU Horizon 2020 project: Factor structure and psychometric properties of the Resilience Scale for Adolescents. Front Psychol. 2021;12:629357. https://doi.org/10.3389/fpsyg.2021.629357

Aro HM. Loneliness and its association with well-being in university students. J Soc Clin Psychol. 2018;37(5):352-69.

Bifulco A, Bernazzani O, Moran PM, Jacobs C. The childhood experience of care and abuse questionnaire (CECA.Q): Validation in a community series. Br J Clin Psychol. 2005;44(4):563-81. https://doi.org/10.1348/014466505X35344

Bifulco A, Brown GW, Harris TO. Childhood Experience of Care and Abuse (CECA): a retrospective interview measure. J Child Psychol Psychiatry. 1994;35(8):1419-35. https://doi.org/10.1111/j.1469-7610.1994.tb01284.x

Brodsky BS, et al. The Childhood Experience of Care and Abuse (CECA): A retrospective measure of early trauma. J Affect Disord. 2001;62(3):249-59.

Bush K, Kivlahan DR, McDonell MB, Fihn SD, Bradley KA. The AUDIT Alcohol Consumption Questions (AUDIT-C): An effective brief screening test for problem drinking. Arch Intern Med. 1998;158(16):1789-95. https://doi.org/10.1001/archinte.158.16.1789

Bush K, Kivlahan DR, McDonnell MB, Fihn SD, Bradley KA. The Alcohol Use Disorders Identification Test (AUDIT): Validation of a screening instrument for use in medical settings. JAMA. 1998;280(2):179-86. https://doi.org/10.1001/jama.280.2.179

Cappuccio FP, D'Elia L, Strazzullo P, Miller MA. Sleep duration and all-cause mortality: A systematic review and meta-analysis of prospective studies. Sleep. 2018;31(5):585-92. https://doi.org/10.1093/sleep/31.5.585

Clarke A, Friede T, Putz R, Ashdown J, Martin S, Blake A, et al. Warwick-Edinburgh Mental Well-being Scale (WEMWBS): Validated for teenage school students in England and Scotland. A mixed methods assessment. BMC Public Health. 2011;11(1). https://doi.org/10.1186/1471-2458-11-487

Clement S, Brohan E, Jeffery D, Henderson C, Hatch SL, Thornicroft G. Development and psychometric properties of the Barriers to Access to Care Evaluation scale (BACE) related to people with mental ill health. BMC Psychiatry. 2012;12:36. https://doi.org/10.1186/1471-244X-12-36

Cohen S, Kamarck T, Mermelstein R. A Global Measure of Perceived Stress. J Health Soc Behav. 1983;24(4):385. https://doi.org/10.2307/2136404

Cotton MA, Ball C, Robinson P. Four simple questions can help screen for eating disorders. J Gen Intern Med. 2003;18:53-6. https://doi.org/10.1046/j.1525-1497.2003.20374.x

Dixon E, Ferris K, Roby T. Assessing student engagement: Validation of the College Student Subjective Wellbeing Questionnaire. J Educ Psychol. 2020;112(4):672-84. https://doi.org/10.1037/edu0000437

Doğan T, Çötok NA, Tekin EG. Reliability and validity of the Turkish Version of the UCLA Loneliness Scale (ULS-8) among university students. Procedia Soc Behav Sci. 2011;15:2058-62. https://doi.org/10.1016/j.sbspro.2011.04.053

Espie CA, Kyle SD, Hames P, Gardani M, Fleming L, Cape J. The Sleep Condition Indicator: A clinical screening tool to evaluate insomnia disorder. BMJ Open. 2014;4(3):e004183. https://doi.org/10.1136/bmjopen-2013-004183

Extremera N, Fernandez-Berrocal P. Validity and reliability of Spanish versions of the ruminative responses scale-short form and the distraction responses scale in a sample of Spanish high school and college students. Psychol Rep. 2006;98:141-50. https://doi.org/10.2466/pr0.98.1.141-150

Fonseca-Pedrero E, Ortuño-Sierra J, Chocarro E, Inchausti F, Debbané M, Bobes J. Psychosis risk screening: Validation of the youth psychosis at-risk questionnaire-brief in a community-derived sample of adolescents. Int J Methods Psychiatr Res. 2017;26(4):e1543. https://doi.org/10.1002/mpr.1543

Gratz KL, Roemer L. Multidimensional Assessment of Emotion Regulation and Dysregulation: Development, Factor Structure, and Initial Validation of the Difficulties in Emotion Regulation Scale. J Psychopathol Behav Assess. 2004;26(1):41-54. https://doi.org/10.1023/b:joba.0000007455.08539.94

Hayes JA, Lockard AJ, Janis RA, Locke BD. Construct validity of the Self-Compassion Scale-Short Form among psychotherapy clients. Couns Psychol Q. 2016;29(4):405-22.

He J, Liu Y, Cheng C, Fang S, Wang X, Yao S. Psychometric Properties of the Chinese Version of the 10-Item Ruminative Response Scale Among Undergraduates and Depressive Patients. Front Psychiatry. 2021;12:626859. https://doi.org/10.3389/fpsyt.2021.626859

Hjemdal O, Friborg O, Stiles TC, Martinussen M, Rosenvinge JH. A new scale for adolescent resilience: Grasping the central protective resources behind healthy development. Meas Eval Couns Dev. 2006;39(2):84-96.

Hughes ME, Waite LJ, Hawkley LC, Cacioppo JT. A short scale for measuring loneliness in large surveys. Res Aging. 2004;26(6):655-72.

Ivtzan I, Young T, Lee HC, Lomas T, Daukantaitė D, Kjell ONE. Mindfulness based flourishing program: a cross-cultural study of Hong Kong Chinese and British participants. J Happiness Stud. 2018;19(8):2205-23. https://doi.org/10.1007/s10902-017-9919-1

Janousch C, Anyan F, Hjemdal O, Hirt CN. Psychometric properties of the resilience scale for adolescents (READ) and measurement invariance across two different German-speaking samples. Front Psychol. 2020;11:608677. https://doi.org/10.3389/fpsyg.2020.608677

Keane LM, Heron J. Assessing cognitive and behavioral responses to stress in adolescents: A psychometric evaluation of the CB-RSS. J Clin Child Adolesc Psychol. 2020;49(1):112-23. https://doi.org/10.1080/15374416.2019.1701092

Keane M, Thomas K, Greenfield D. Reliability and validity of the Cognitive and Behavioral Response to Stress Scale in adolescents. J Behav Med. 2020;43(5):643-50. https://doi.org/10.1007/s10865-020-00135-7

Kechter A, Black DS, Riggs NR. Developmental trajectories of perceived stress and coping among ethnic minority adolescents during the transition to high school. Int J Environ Res Public Health. 2019;16(9):1609. https://doi.org/10.3390/ijerph16091609

Kotera Y, Green P, Sheffield D. Mental health shame of UK construction workers: Relationship with masculinity, work motivation, and self-compassion. J Work Organ Psychol. 2019;35(2):135-43. https://doi.org/10.5093/jwop2019a15

Kroenke K, Spitzer RL, Williams JB. The PHQ-9: Validity of a brief depression severity measure. J Gen Intern Med. 2001;16(9):606-13.

Lasgaard M. Reliability and validity of the Danish version of the UCLA Loneliness Scale. Pers Individ Dif. 2007;42(7):1359-66. https://doi.org/10.1016/j.paid.2006.10.013

Lee EH. Review of the psychometric evidence of the Perceived Stress Scale. Asian Nurs Res. 2012;6(4):121-7. https://doi.org/10.1016/j.anr.2012.08.004

Lee S, Kim W. Cross-cultural adaptation, reliability, and validity of the revised Korean version of ruminative response scale. Psychiatry Investig. 2014;11:59-64. https://doi.org/10.4306/pi.2014.11.1.59

Levis B, et al. The PHQ-9 depression scale: A comprehensive review. J Affect Disord. 2019;256:191-8.

Linden B, Stuart H. Psychometric assessment of the Post-Secondary Student Stressors Index (PSSI). BMC Public Health. 2019;19:1139. https://doi.org/10.1186/s12889-019-7472-z

Liu W, Wei H, Zhao S, Wang X. Perceived stress among Chinese adolescents: Measurement invariance of the Perceived Stress Scale and relationships with mental health. Psychol Res Behav Manag. 2020;13:391-401. https://doi.org/10.2147/PRBM.S246189

Löwe B, et al. The GAD-7 as a screening tool for anxiety disorders in the general population. J Affect Disord. 2008;111(1):137-44.

Meulenbeek P, Brouwers A, Koppes L. The Cognitive and Behavioral Response to Stress Scale (CB-RSS): Psychometric properties and its application in stress research. J Stress Health. 2017;33(4):450-8. https://doi.org/10.1002/johs.1236

Meulenbeek PA, van der Wal RC, Engels RC. The Cognitive and Behavioral Response to Stress Scale (CB-RSS): A validation study in adolescents. J Youth Adolesc. 2017;46(4):765-78. https://doi.org/10.1007/s10964-017-0673-3

Miner AG, Bowers ME, Bowers JW. Creation and validation of the Cognitive and Behavioral Response to Stress Scale (CB-RSS). J Psychopathol Behav Assess. 2015;37(3):456-68. https://doi.org/10.1007/s10862-015-9503-2

Morgan JF, Reid F, Lacey JH. The SCOFF questionnaire: a new screening tool for eating disorders. West J Med. 2000;172(3):164.

Parker SC, Lyons J, Bonner J. Eating disorders in graduate students: exploring the SCOFF questionnaire as a simple screening tool. J Am Coll Health. 2005;54:103-7. https://doi.org/10.3200/JACH.54.2.103-107

Phalen PL, Rouhakhtar PR, Millman ZB, Thompson E, DeVylder J, Mittal V, et al. Validity of a two-item screen for early psychosis. Psychiatry Res. 2018;270:861-8. https://doi.org/10.1016/j.psychres.2018.11.002

Posner K, et al. The Columbia-Suicide Severity Rating Scale: Initial validity and internal consistency findings from the web-based suicide risk assessment in adolescent and adult psychiatric patients. J Clin Psychiatry. 2011;72(9):1306-17.

Raes F, Pommier E, Neff KD, Van Gucht D. Construction and factorial validation of a short form of the Self-Compassion Scale. Clin Psychol Psychother. 2010;18(3):250-5. https://doi.org/10.1002/cpp.702

Reinert DF, Allen JP. The Alcohol Use Disorders Identification Test: An update of research findings. Alcohol Clin Exp Res. 2007;31(2):185-99. https://doi.org/10.1111/j.1530-0277.2006.00295.x

Renshaw TL, Bolognino SJ. The College Student Subjective Wellbeing Questionnaire: A brief, multidimensional measure of undergraduate’s covitality. J Happiness Stud. 2016;17(2):463-84. https://doi.org/10.1007/s10902-015-9606-4

Renshaw TL. College Student Subjective Wellbeing Questionnaire (CSSWQ): measure and user guide. Open Science Framework. 2022. https://doi.org/10.17605/OSF.IO/MJRKP

Richardson LA, et al. The PHQ-9 as a measure of depression in university students. J Coll Stud Psychother. 2010;24(3):207-21.

Rodríguez-Rey R, Alonso-Tapia J, Hernansaiz-Garrido H. Reliability and validity of the Brief Resilience Scale (BRS) Spanish version. Psychol Assess. 2016;28(5):e101-e110. https://doi.org/10.1037/pas0000191

Schoofs H, Hermans D, Raes F. Brooding and reflection as subtypes of rumination: evidence from confirmatory factor analysis in nonclinical samples using the Dutch ruminative response scale. J Psychopathol Behav Assess. 2010;32:609-17. https://doi.org/10.1007/s10862-010-9182-9

Serrani Azcurra D. Psychometric validation of the Columbia-Suicide Severity rating scale in Spanish-speaking adolescents. Colomb Med (Cali). 2017;48(4):174-82.

Smith BW, Dalen J, Wiggins K, Tooley E, Christopher P, Bernard J. The Brief Resilience Scale: Assessing the ability to bounce back. Int J Behav Med. 2008;15(3):194-200. https://doi.org/10.1080/10705500802222972

Smith BW, Epstein EM, Ortiz JA, Christopher P, Tooley EM. The foundations of resilience: What are the critical resources for bouncing back from stress? In: Prince-Embury S, Saklofske DH, editors. Resilience in children, adolescents, and adults: Translating research into practice. New York: Springer; 2013. p. 167-87.

Spitzer RL, Kroenke K, Williams JB, Löwe B. A brief measure for assessing generalized anxiety disorder: The GAD-7. Arch Intern Med. 2006;166(10):1092-7.

Taylor S, Landry CA, Paluszek MM, Fergus TA, McKay D, Asmundson GJG. Development and initial validation of the COVID Stress Scales. J Anxiety Disord. 2020;72:102232. https://doi.org/10.1016/j.janxdis.2020.102232

Tennant R, Hiller L, Fishwick R, Platt S, Joseph S, Weich S, et al. The Warwick-Edinburgh mental well-being scale (WEMWBS): development and UK validation. Health Qual Life Outcomes. 2007;5(1):63.

Treynor W, Gonzalez R, Nolen-Hoeksema S. Rumination reconsidered: a psychometric analysis. Cognit Ther Res. 2003;27(3):247-59. https://doi.org/10.1037/t65937-000

Tyrka AR, et al. Childhood maltreatment and adult personality disorders: A review of the literature. J Pers Disord. 2008;22(4):347-69.

Vanheusden K, Mulder CL, van der Ende J, van Lenthe FJ, Mackenbach JP, Verhulst FC. Young adults face major barriers to seeking help from mental health services. Patient Educ Couns. 2008;73(1):97-104. https://doi.org/10.1016/j.pec.2008.05.006

Victor SE, Klonsky ED. Validation of a brief version of the Difficulties in Emotion Regulation Scale (DERS-18) in five samples. J Psychopathol Behav Assess. 2016;38(4):582-9. https://doi.org/10.1007/s10862-016-9547-9

Von Soest T, Mossige S, Stefansen K, Hjemdal O. A validation study of the Resilience Scale for Adolescents (READ). J Psychopathol Behav Assess. 2010;32(2):215-25. https://doi.org/10.1007/s10862-009-9149-x

Wheaton MG, Ward HE, Sierra MA, Abramowitz JS. Pandemic-related stress and anxiety: Associations with depression, anxiety, and quality of life. Psychol Med. 2021;51(10):1-10. https://doi.org/10.1017/S0033291721000934

Zhang YL, Liang W, Chen ZM, et al. Validity and reliability of Patient Health Questionnaire-9 and Patient Health Questionnaire-2 to screen for depression among college students in China. Asia Pac Psychiatry. 2013;5:268-75.
