# Supplementary material for: Nurture-U student mental health longitudinal survey: a study protocol
Source: BMJ Open. 2025 Feb 11;15(2):e098413. doi: 10.1136/bmjopen-2024-098413 (PMC11815430; doi:10.1136/bmjopen-2024-098413)
Supplement: Supplementary file 2 [file bmjopen-2024-098413.reviewer_comments.pdf]

PEER REVIEW HISTORY

BMJ Open publishes all reviews undertaken for accepted manuscripts. Reviewers are asked to complete a checklist review form and are provided with free text boxes to elaborate on their assessment. These free text comments are reproduced below.

ARTICLE DETAILS

Title (Provisional)

Nurture-U student mental health longitudinal survey: a study protocol

Authors

Marshall, Ellen; Duffy, Anne C; Chamberlain, Samuel R.; Dooley, Jemima; Dorey, Lucy; Forty, Liz; Matlock, Kevin A.; Newbold, Alexandra; Quinn, Anthony; Rees, Sarah; Robinson, Lucy; Saunders, Kate E; Watkins, Edward

---

This article was not externally reviewed at BMJ Open. Protocols that have been independently assessed prior to submission to BMJ Open are usually fast-tracked to publication on the grounds that further substantial changes will not be possible. This independent assessment will usually be external, independent review for both a substantial grant award from a non-commercial or government funder and ethics approval.
